# Vision-Based Human–Computer Interaction System for Mexican Sign Language Recognition and Robotic Reproduction: Toward Inclusive Educational Applications

**DOI:** 10.3390/s26216990

**Published:** 2026-11-05

**Authors:** David Alexis Cano-Ibarra, Wendy Flores-Carmona, Karen Roa-Tort, Diego A. Fabila-Bustos, Macaria Hernández-Chávez, J. Alejandro Aguirre-Anaya, Josué D. Rivera-Fernández

**Affiliations:** 1 Laboratorio de Optomecatrónica, UPIIH, Instituto Politécnico Nacional, Distrito de Educación, Salud, Ciencia, Tecnología e Innovación, San Agustín Tlaxiaca 42162, Mexico; dcanoi2000@alumno.ipn.mx (D.A.C.-I.); wfloresc1700@alumno.ipn.mx (W.F.-C.); kroat@ipn.mx (K.R.-T.); dfabilab@ipn.mx (D.A.F.-B.); mhernandezch@ipn.mx (M.H.-C.); 2 Unidad Profesional Interdisciplinaria de Energía y Movilidad, Instituto Politécnico Nacional, Av. Wilfrido Massieu, Adolfo López Mateos S/N, Nueva Industrial Vallejo, Gustavo A. Madero, Mexico City 07738, Mexico; jaguirrea@ipn.mx

**Keywords:** human–computer interaction, mexican sign language, sign language recognition, computer vision, hand landmark detection, machine learning, robotic interaction, inclusive education

## Abstract

Mexican Sign Language (MSL) plays an essential role in communication within the Deaf community, yet access to sign-language learning resources remains limited in some educational settings. This study presents a vision-based Human–Computer Interaction (HCI) system for MSL recognition and robotic reproduction, developed as a proof of concept toward inclusive educational applications. The system uses an RGB camera and MediaPipe Hands for hand-landmark extraction, a multilayer perceptron (MLP) for recognition of 29 static classes, and a TimeDistributed–Long Short-Term Memory (LSTM) model for 10 dynamic classes. Recognized signs are mapped to predefined motion sequences executed by an ATmega2560-controlled robotic arm. Under stratified random and sequential partitioning protocols, the static model achieved accuracies of 100.00% and 99.88%, respectively, while the dynamic model achieved 100.00% under both protocols. Additional comparisons with Support Vector Machine (SVM) and Random Forest classifiers, using three random seeds, demonstrated competitive performance and low variability within the original single-participant dataset. To assess generalization beyond the training participant, a preliminary signer-independent evaluation was conducted with three previously unseen participants, without model retraining or adaptation. Across 585 recognition attempts involving 39 classes, the system achieved an overall accuracy of 89.06%, providing initial evidence of recognition performance across different signers. Furthermore, 92 accepted recognition trials successfully triggered their corresponding robotic sequences, demonstrating functional integration between recognition and actuation. These findings support the technical feasibility of the proposed contactless HCI architecture as a potential foundation for interactive MSL learning tools. Nevertheless, broader signer-independent validation and user-centered evaluations are required to establish its accessibility, usability, and educational effectiveness.

## 1. Introduction

Human–Computer Interaction (HCI) has increasingly incorporated natural and accessible interaction modalities that reduce the need for conventional physical interfaces. In this context, vision-based interaction enables users to communicate with computational systems through natural body movements and gestures, providing a contactless interaction mechanism that can be particularly relevant for assistive and educational applications. For Deaf and hard-of-hearing communities, sign languages constitute a natural communication modality; therefore, their integration into vision-based HCI systems represents an opportunity to develop more accessible interfaces capable of interpreting human gestures without requiring specialized wearable devices or instrumented gloves. This need is particularly relevant given the large number of people worldwide who experience hearing loss and the persistent communication barriers between Deaf and hearing communities.

According to the World Health Organization (WHO), more than 1.5 billion people worldwide live with hearing loss, including approximately 430 million with disabling hearing loss who require rehabilitation. This number is projected to reach nearly 2.5 billion by 2050 [1]. For a large portion of the Deaf population, sign language represents a primary means of communication; however, most hearing individuals lack proficiency in sign languages, creating persistent communication barriers between Deaf and hearing communities, particularly in educational settings [2]. In Mexico, more than four million individuals experience communication barriers associated with hearing impairment, while an estimated 60% of the Deaf population remains illiterate, largely due to limited access to bilingual education [2]. This situation is not primarily attributable to the technological infeasibility or legal recognition of sign language—Mexican Sign Language (MSL) has held official national-language status since 2005—but rather to the limited availability of trained bilingual educators and interpreters relative to the size of the Deaf student population [3]. Consequently, the development of accessible technological interfaces capable of recognizing MSL offers a complementary approach to existing educational and communication resources.

Automatic sign language recognition (SLR) has traditionally been approached through two main technological paradigms: wearable sensing systems and computer-vision-based systems [4,5]. Wearable approaches commonly employ flex sensors, inertial measurement units (IMUs), or combinations of both to directly capture finger flexion, hand orientation, and hand motion. The acquired signals can be processed using microcontroller-based platforms such as Arduino or ESP32 and subsequently analyzed using machine-learning or deep-learning architectures, including LSTM and hybrid CNN-RNN models [4]. Recent developments have also explored alternative wearable sensing technologies, such as Hall-effect sensors, to reduce mechanical degradation associated with conventional flex sensors. For example, Padhya et al. developed a low-cost sign-language glove based on Hall-effect sensors and an IMU, with an embedded multilayer perceptron implemented on an Arduino Mega, achieving 96% recognition accuracy for a fundamental Nepali Sign Language vocabulary [6]. Recognition accuracies ranging from approximately 85% to 99% have been reported for static alphabet recognition, while wearable systems can offer reduced sensitivity to illumination and background variations [5]. Nevertheless, the requirement for physical devices worn by the user introduces additional interaction requirements that may affect comfort, usability, scalability, and adoption. Mechanical degradation of sensing elements and the additional cost associated with wearable hardware may also limit their suitability for widespread deployment in educational environments [5,6,7].

Computer-vision-based approaches provide an alternative interaction modality by using cameras as the primary sensing interface and eliminating the need for instrumented gloves or other wearable devices. RGB and depth cameras have been employed to capture information from the hands, body, and face, while computer-vision algorithms transform the acquired visual information into representations suitable for machine-learning classification. This configuration is particularly relevant to HCI because it allows the user’s natural gestures to constitute the input modality without requiring direct physical contact with the sensing system.

Among the available computer-vision frameworks, MediaPipe Hands has been widely adopted for hand-landmark extraction and provides 21 three-dimensional landmarks per detected hand from RGB images [8]. Landmark-based representations offer a compact description of hand configuration and movement compared with approaches that directly process complete RGB frames. These representations can subsequently be combined with machine-learning models for static recognition or temporal architectures for dynamic gestures [9,10]. Recent studies have employed hand landmarks together with temporal classifiers, including LSTM-based models, to recognize fingerspelling letters and dynamic sign sequences [9,10]. Such approaches demonstrate the feasibility of using compact visual representations to reduce the dimensionality of the classification problem while preserving relevant spatial and temporal information.

Recent developments in machine learning have further expanded the computational platforms on which vision-based sign recognition can be implemented. Attention-enhanced LSTM architectures deployed on resource-constrained platforms have achieved recognition rates of up to 99.7%, while hybrid LSTM and three-dimensional convolutional neural network architectures have reported F1 scores above 0.93 in computationally constrained settings [11,12,13]. Other studies have explored TinyML, quantized models, and lightweight object-detection architectures to reduce computational requirements and facilitate deployment on edge platforms [11,14]. These approaches demonstrate the increasing maturity of machine-learning-based sign recognition and the possibility of implementing visual recognition under different computational constraints. However, the use of edge or embedded platforms is not, by itself, sufficient to establish a complete HCI system; the interaction architecture must also consider how recognized information is communicated to the user or translated into an actionable output.

An additional consideration in vision-based sign-language recognition is the type of visual information used by the recognition model. Although hand landmarks provide an efficient representation, sign languages involve more than hand configuration. Body posture, spatial relationships, and facial expressions may also contribute to the linguistic content of a sign. Studies specifically addressing MSL have investigated RGB-D approaches that capture hand, body, and facial landmarks for static and dynamic signs [15,16]. These works indicate that hand-only representations constitute a simplification of the complete visual structure involved in sign-language communication. In the present study, hand-based recognition is deliberately adopted as an initial HCI modality, while the incorporation of body and facial information is considered a relevant direction for future development.

The development of datasets specifically focused on MSL has also contributed to the advancement of computational recognition systems. The MX-ITESO-100 lexical video dataset [17] provides a reference for machine-learning research involving commonly used Mexican Sign Language signs and was developed through a community-oriented selection process. Other investigations of MSL have examined word-level recognition using RGB-D information [17]. Related work on American Sign Language (ASL) has investigated neural-network-based approaches, including autoencoder architectures trained using sign-language images acquired with depth sensors [18]. These studies provide relevant methodological context for computational sign-language recognition, while the development of MSL-specific systems requires consideration of the linguistic and representational characteristics of MSL.

Beyond recognition accuracy, sign-language technologies can be understood from an HCI perspective as interactive systems in which visual sensing establishes a communication channel between the human user and a computational interface. From this perspective, the camera is not merely an image-acquisition device but the primary sensing interface through which natural human gestures are converted into computational information. Recognition consequently represents one stage of the interaction process rather than its complete outcome. A more comprehensive interaction architecture requires the recognized information to generate a meaningful response, which may be visual, auditory, haptic, or physical.

Robotic actuation provides an additional physical feedback modality for sign-language interaction and learning. Robotic hands capable of reproducing manual alphabets from voice commands or predefined configurations have been reported, including implementations on low-cost computational platforms such as the Raspberry Pi 4, with reproduction accuracies approaching 94% [19]. Open-source robotic hands with multiple servomotors have also been developed to reproduce configurations associated with the American Sign Language alphabet [20]. Other approaches have investigated perception-to-action mappings using depth sensors and humanoid robotic systems, allowing robotic platforms to reproduce sign motions derived from human demonstrations rather than relying exclusively on individually programmed configurations [21]. These studies demonstrate the feasibility of robotic sign reproduction; however, robotic generation and visual sign recognition have frequently been investigated as separate technological components.

This separation represents an important opportunity from an HCI perspective. A recognition system evaluated exclusively through classification accuracy demonstrates whether a computational model can identify a gesture, but it does not necessarily demonstrate how that information contributes to interaction with a user. Conversely, a robotic system capable of reproducing predefined signs does not necessarily establish a complete perception-to-action interaction loop. Integrating visual sensing, machine-learning-based interpretation, and robotic response can therefore establish a broader interaction architecture in which a natural human gesture is sensed, computationally interpreted, and transformed into an observable physical response.

Despite the advances described above, relatively few approaches appear to integrate vision-based MSL recognition with robotic physical feedback within a unified HCI architecture. Existing MSL studies have primarily focused on visual recognition and classification [15,17], while robotic sign-generation systems have generally addressed physical reproduction using predefined configurations or independent input modalities [19,20,21]. Similarly, previous vision-based approaches have demonstrated high recognition performance using landmark representations and temporal models [8,9,10], but the recognition stage is commonly treated as the final output of the system. This suggests an opportunity to investigate an HCI architecture in which sign recognition is directly connected to a physical robotic response.

To address this gap, this work proposes a vision-based Human–Computer Interaction system for Mexican Sign Language recognition and robotic reproduction. An RGB camera is employed as the primary sensing interface, while MediaPipe Hands is used to extract three-dimensional hand landmarks that characterize the user’s gestures [13]. Two complementary machine-learning branches are implemented according to the temporal characteristics of the signs: a multilayer perceptron (MLP) for static signs and a TimeDistributed–Long Short-Term Memory (LSTM) based architecture for dynamic signs. Following recognition, the resulting class label is associated with a predefined sequence of robotic joint configurations and transmitted via UART to an ATmega2560 microcontroller, which controls the robotic arm. The resulting architecture establishes an interaction loop between the human user, the visual sensing interface, the computational recognition system, and the robotic actuator.

The proposed approach is motivated not only by the technological challenge of MSL recognition but also by the need for accessible interaction technologies that can complement existing educational resources. In this regard, the work is conceptually aligned with Sustainable Development Goal 4 (SDG 4: Quality Education), particularly through the development of technologies that may support inclusive and accessible learning environments, and with Sustainable Development Goal 10 (SDG 10: Reduced Inequalities), through technological approaches aimed at reducing communication barriers between Deaf and hearing communities. The proposed system is therefore considered a technological foundation for future user-centered educational applications rather than a direct assessment of educational or social impact.

The contribution of this work does not lie in introducing a new hand-landmark extraction method or a novel recognition algorithm. Instead, the proposed approach integrates established computer-vision and machine-learning techniques into a unified interaction pipeline for Mexican Sign Language (MSL). MediaPipe Hands is used for landmark extraction, an MLP is employed for static sign classification, and a TimeDistributed–LSTM-based architecture is used for dynamic sign recognition. These components are connected to a deterministic robotic actuation layer that translates recognized signs into predefined robotic responses. Thus, the technical contribution lies primarily in the integration of MSL visual recognition with physical robotic feedback within a single interaction pathway, together with its experimental evaluation under different data-partitioning protocols.

The main contributions of this work are summarized as follows: (i) a proof-of-concept, vision-based HCI architecture in which Mexican Sign Language (MSL) is employed as a natural, contactless interaction modality; (ii) an integrated dual recognition strategy for static and dynamic MSL signs, using established MLP and LSTM-based models, respectively, according to the different spatial and temporal characteristics of the signs; (iii) a recognition-to-actuation interface that integrates visual sign recognition with predefined robotic responses, establishing a unified interaction pathway from gesture acquisition and recognition to physical robotic feedback; (iv) an experimental evaluation of the integrated recognition pipeline using random and sequential data-partitioning protocols, complemented by comparisons with alternative classifiers and repeated experiments to assess statistical variability; and (v) a discussion of the prototype’s potential relevance to inclusive education and accessibility, with conceptual alignment with Sustainable Development Goals (SDGs) 4 and 10.

The remainder of this article is organized as follows. Section 2 describes the proposed system, including the hardware platform, vision-based HCI pipeline, feature extraction and classification models, and recognition-to-actuation mechanism. Section 3 describes the experimental evaluation and reports the obtained results. Section 4 discusses recognition performance, the relationship between the proposed approach and existing methods, the limitations of the system, and its potential relevance to inclusive educational applications and Sustainable Development Goals (SDGs) 4 and 10. Finally, Section 5 presents the conclusions and future research directions.

## 2. Materials and Methods

### 2.1. System Architecture and Hardware Platform

The proposed system consists of three hardware blocks with distinct functional responsibilities: an RGB camera for visual acquisition, a host computer responsible for image processing and machine-learning inference, and an ATmega2560 microcontroller dedicated exclusively to robotic actuation. This architecture establishes a vision-based Human–Computer Interaction (HCI) pipeline in which the user’s hand gestures constitute the input modality and the robotic arm provides a physical response.

The RGB camera constitutes the primary sensing interface. Image acquisition and frame processing are performed on the host computer using OpenCV. The same computer executes the MediaPipe Hands framework for hand-landmark extraction and the corresponding machine-learning model for sign classification. Depending on the selected operating mode, either the static MLP or the dynamic TimeDistributed–LSTM-based model is executed.

The system was implemented on a host computer equipped with an AMD Ryzen 5 8645HS processor (Advanced Micro Devices, Inc., Santa Clara, CA, USA). with integrated Radeon 760M Graphics, 32 GB of RAM, and an NVIDIA GeForce RTX 4050 GPU with 6 GB of dedicated memory, running Windows 11. The software was developed in Python 3.9 using Visual Studio Code (VS Code, version 1.96.0) as the development environment. TensorFlow 2.16.1 and Keras 3.14.1 were used to implement the neural-network models, while OpenCV 4.10.0.84 and MediaPipe 0.10.14 were employed for image acquisition and processing, and hand-landmark extraction, respectively.

The trainable parameters of both recognition models were initialized using the default Keras initialization schemes. Dense-layer weights were initialized using Glorot uniform initialization, whereas the LSTM layers employed Glorot uniform initialization for input weights and orthogonal initialization for recurrent weights. Bias parameters followed the default Keras settings, including the unit forget-gate bias configuration in the LSTM layers. No custom weight-initialization procedure was applied. Training was performed for a fixed number of epochs, with 100 epochs for the static MLP and 150 epochs for the dynamic TimeDistributed–LSTM model. No early-stopping mechanism was employed, and training was terminated upon completion of the predefined number of epochs.

The distribution of computational tasks in the proposed system differs from fully embedded sign-language recognition architectures reported in previous studies, in which the inference stage is implemented on platforms such as Raspberry Pi, Jetson Nano, or resource-constrained microcontrollers [5,6,7]. In the present implementation, both landmark extraction and classification are performed on the host computer. The ATmega2560 does not participate in visual processing or sign recognition. Instead, after a sign has been classified, the host computer retrieves the corresponding predefined robotic motion and transmits the resulting joint-angle sequence to the microcontroller through a UART serial connection configured at 9600 baud.

The ATmega2560 is therefore dedicated exclusively to actuation. It receives the joint-angle commands generated by the host computer and sequentially applies them to the servomotors of the robotic arm. The arm reproduces the predefined sequence until the corresponding sign motion is completed, after which the system returns the mechanism to its predefined resting configuration.

This architecture provides a clear separation between perception, recognition, and actuation. The host computer performs computationally intensive image-processing and machine-learning tasks, whereas the microcontroller provides the low-level control required for robotic actuation. Consequently, the current implementation should not be considered a fully embedded vision system. Migration of the visual-processing and inference stages to a suitable edge-computing platform represents a potential direction for future development, particularly for applications requiring greater portability and autonomous operation.

### 2.2. Vision-Based HCI Pipeline and Data Flow

The vision-based interaction pipeline follows a sequential processing architecture composed of five main stages: (1) image acquisition using OpenCV and hand-landmark extraction using MediaPipe Hands, (2) manual selection of the operating mode, (3) inference using the corresponding classification model, (4) prediction evaluation based on confidence and cooldown conditions, and (5) recognition-to-actuation mapping followed by robotic reproduction.

The operating mode is selected manually by the user accor and the normalized coordinates ding to the type of sign to be recognized. In static mode, the system analyzes individual frames, whereas dynamic mode processes temporal sequences of consecutive frames. This manual selection was adopted to explicitly distinguish between static hand configurations and signs whose meaning depends on temporal movement.

An OpenCV-based graphical interface provides visual feedback during system operation. The interface includes a heads-up display (HUD) containing the active recognition mode, the most recently accepted gesture, the corresponding confidence value, the UART communication status, and a cooldown progress indicator. These elements allow the operator to monitor the state of the HCI system and inspect the information being generated during interaction. Figure 1 illustrates the graphical interface used during system operation and shows the principal status elements provided by the HUD.

The complete data flow, from image acquisition to robotic actuation, can be represented by five transformation stages.

Stage 1—Image acquisition and landmark detection. Each frame captured by the RGB camera is processed using MediaPipe Hands. For each detected hand, the framework provides 21 three-dimensional landmarks representing the spatial configuration of the hand. The system is configured to process up to two hands simultaneously.

Stage 2—Feature vectorization and normalization. The three-dimensional coordinates of the detected landmarks are concatenated to generate a fixed-dimensional representation. When two hands are detected, the resulting vector contains 126 values, corresponding to 21 landmarks with three coordinates per landmark for each of the two hands.

The coordinates are normalized relative to the wrist position to reduce the influence of the absolute location of the hands within the image. Specifically, for each landmark *P_i_* = (*x_i_*,*y_i_*,*z_i_*), the normalized coordinates are computed with respect to the wrist landmark *P_w_* = (*x_w_*,*y_w_*,*z_w_*) as $P_{i}^{\prime }=P_{i}-P_{w}$. Where *P_i_* denotes landmark *i*, and *P_w_* denotes the wrist landmark.

Thus, the wrist landmark is mapped to the origin, $P_{w}^{\prime }=(0,0,0)$, while the relative spatial configuration of the hand is preserved. This transformation reduces sensitivity to global hand translation. More specifically, if all landmarks undergo the same translation (*T*) then, $P_{i}^{*}=P_{i}+T,P_{w}^{*}=P_{w}+T,$ and the normalized coordinates become $P_{i}^{\prime *}=P_{i}^{*}-P_{w}^{*}=\left(P_{i}+T\right)-\left(P_{w}+T\right)=P_{i}-P_{w}=P_{i}^{\prime }$, where ${P_{i}}^{*\prime }$ denotes the landmark *i* translation.

Therefore, the normalized representation is invariant to global translations of the hand. When only one hand is detected, the corresponding portion of the feature vector associated with the missing hand is filled with zeros, maintaining a fixed input dimensionality.

Stage 3—Conditional temporal buffering. The processing strategy depends on the selected recognition mode. In static mode, each 126-value feature vector is directly provided to the MLP classifier. In dynamic mode, consecutive feature vectors are accumulated into a temporal sequence until the predefined sequence length is reached. At that point, the complete buffer (a matrix with dimensions of 30 frames × 126) is sent to the LSTM model.

Stage 4—Classification and decision evaluation. The selected classification model produces a probability distribution over the corresponding sign classes. Let *p_c_* denote the predicted probability associated with class (c). During system operation, a prediction is accepted only if its maximum class probability exceeds the confidence threshold of 0.85 and the predefined cooldown period since the previous accepted prediction has elapsed. Predictions that do not satisfy these conditions are discarded, and the system continues processing subsequent frames. For the model-level evaluation, all predictions generated for the evaluation datasets were considered, regardless of their confidence value or subsequent acceptance by the threshold and cooldown criteria. Therefore, the confusion matrices and the reported recognition metrics reflect all model predictions and were calculated before applying the confidence threshold and cooldown mechanism.

Stage 5—Recognition-to-actuation mapping. Once a sign has been accepted, its class label is provided to a deterministic mapping module. The module retrieves the predefined sequence of robotic joint configurations associated with the recognized class. The resulting joint-angle configurations are serialized and transmitted sequentially through UART to the ATmega2560. Communication is executed independently from the primary image-acquisition loop so that robotic actuation does not unnecessarily interrupt the visual-processing pipeline.

The complete interaction chain can therefore be summarized as:

- Human gesture;
- RGB image;
- Hand landmarks;
- Feature vector;
- ML classification;
- MSL class;
- Joint-angle sequence;
- Robotic response.

This processing chain establishes the proposed HCI mechanism by connecting a natural human gesture to a computational interpretation and, subsequently, to a physical robotic response.

### 2.3. Feature Extraction and Classification Models

#### 2.3.1. Hand-Landmark Feature Extraction

Unlike image-based approaches that directly process complete RGB frames using convolutional neural networks, the proposed system employs MediaPipe Hands as an intermediate visual representation. The framework detects 21 three-dimensional landmarks for each hand, providing a compact description of hand geometry and configuration. This representation allows subsequent classifiers to operate on a compact vector representation instead of raw image data, while the normalization procedure provides translation invariance, as described in Section 2.2.

#### 2.3.2. Static Sign Recognition

Static signs are classified using a multilayer perceptron (MLP). The model receives a single normalized feature vector containing 126 values and produces a probability distribution over 29 static classes. The 29 classes comprise 21 letters of the MSL fingerspelling alphabet and 8 numerical signs.

The MLP consists of an input layer with 126 features, followed by three fully connected hidden layers containing 512, 256, and 128 neurons, respectively. Rectified Linear Unit (ReLU) activation functions are employed in all three hidden layers. Dropout with a rate of 0.3 (30%) is applied after each hidden layer as a regularization mechanism. Batch normalization is also applied within the hidden layers to improve training stability. The output layer contains 29 neurons, corresponding to the 29 static classes, and uses Softmax activation to obtain the probability distribution over the possible signs.

The MLP was trained using the Adam optimizer with a learning rate of 0.001, a batch size of 32, and a maximum of 100 training epochs. A random seed of 7 was specified to support reproducibility. The selection of the MLP architecture was guided by the structure of the static-sign representation. Since each static sample is represented by a fixed-length vector of 126 normalized landmark features without an explicit temporal dimension, the MLP is used to learn nonlinear relationships among the spatial features for static sign classification.

#### 2.3.3. Dynamic Sign Recognition

Dynamic signs require temporal information because their meaning depends not only on the spatial configuration of the hands but also on their temporal evolution across consecutive frames. For this reason, a TimeDistributed–LSTM-based architecture is employed for dynamic recognition. Each dynamic sample consists of a sequence of 30 consecutive frames, with each frame represented by 126 normalized features, resulting in an input dimension of 30 × 126.

The dynamic model first processes the input sequence through two TimeDistributed Dense layers containing 128 and 64 neurons, respectively. ReLU activation functions are used in both layers, while the TimeDistributed operation independently applies the corresponding transformation to each frame while preserving the temporal dimension. The resulting frame-level representations are subsequently processed by two stacked LSTM layers containing 128 and 64 units, respectively, to model temporal dependencies across consecutive frames. Dropout with a rate of 0.4 (40%) is applied after each LSTM layer as a regularization mechanism. A fully connected layer containing 64 neurons with ReLU activation is then used to further transform the temporal representation. The output layer contains 10 neurons, corresponding to the six letters, two numerical signs, and two words included in the dynamic-sign vocabulary, and uses Softmax activation to produce the probability distribution over the dynamic classes.

The dynamic model was trained using the Adam optimizer with a learning rate of 0.001, a batch size of 32, and a maximum of 150 training epochs. A random seed of 7 was specified to support reproducibility. The selection of the TimeDistributed–LSTM-based architecture was guided by the sequential structure of the dynamic-sign representation. Unlike static signs, dynamic samples contain an explicit temporal dimension consisting of 30 consecutive observations. The TimeDistributed layers independently transform the 126 landmark features at each time step, whereas the subsequent stacked LSTM layers model the temporal evolution of these frame-level representations. This architecture therefore separates the frame-wise feature transformation from the temporal modeling required to characterize dynamic gestures.

### 2.4. Dataset Construction and Evaluation Protocol

The recognition system was evaluated using separate datasets for static and dynamic signs. The static dataset consisted of 29 classes, including 21 letters of the MSL manual alphabet and 8 numerical signs. For 28 classes, 300 samples were collected, whereas 150 samples were available for the letter T. This difference reflects the number of samples collected during the data acquisition process, and no synthetic duplication was introduced to artificially equalize the class sizes. Each static sample corresponded to a single acquired image of the respective hand configuration, resulting in a total of 8550 static samples.

For the static-sign dataset, each sample was an independently acquired image. The 300 samples assigned to each class were not obtained by extracting consecutive frames from a continuous video recording, nor were they generated using overlapping temporal windows. Therefore, the static dataset does not consist of temporally adjacent video frames or overlapping frame sequences.

The dynamic dataset consisted of 10 classes, including 6 letters, 2 numerical signs, and the words “Hola” and “Adiós”. For each class, 75 temporal sequences were collected, resulting in a total of 750 sequences. Each sequence contained 30 consecutive frames acquired at 30 frames per second (fps), corresponding to a temporal span of approximately 1 s per sequence. Each sequence represented an independently acquired execution of the corresponding sign and was acquired independently; therefore, the sequences were not generated by applying overlapping sliding windows to a continuous recording. After completing one acquisition, the same procedure was repeated under different acquisition conditions, including camera viewpoint, background, clothing, and additional visual elements such as caps and eyeglasses. The acquisition of the dynamic classes was performed in alternating order rather than in class-specific blocks, providing variation in acquisition conditions throughout the dataset.

For both static and dynamic datasets, an 80/20 partition was used for training and evaluation. For the static dataset, this resulted in 6840 training samples and 1710 evaluation samples. For the dynamic dataset, the partition was performed at the sequence level, resulting in 600 training sequences and 150 evaluation sequences. Because the dynamic dataset contained 75 sequences per class, the partition corresponded to 60 training sequences and 15 evaluation sequences per class.

Two partitioning protocols were considered to examine the influence of sample partitioning on recognition performance: a stratified random partition and a sequential partition. In the random protocol, complete samples were randomly assigned to the training and evaluation subsets while preserving the class distribution. For the dynamic dataset, each complete 30-frame sequence was treated as the unit of partitioning; consequently, all 30 frames belonging to a given sequence were kept within the same subset. No frames from the same dynamic sequence were distributed between the training and evaluation subsets.

In the sequential protocol, complete samples were arranged according to their original acquisition order, with the first 80% used for training and the final 20% reserved for evaluation. Because the dynamic sequences were acquired in an alternating class order rather than in class-specific blocks, the sequential partition retained samples from the different dynamic classes throughout the acquisition sequence while preserving the original order of acquisition. Thus, the sequential protocol preserves the acquisition order while maintaining sequence-level separation between the training and evaluation subsets.

The original static and dynamic datasets were collected from a single participant under controlled acquisition conditions. Consequently, the stratified random and sequential partitioning protocols described in this section primarily characterize within-subject recognition performance and should not be interpreted as evidence of signer-independent generalization. To complement these experiments, a separate preliminary signer-independent evaluation was subsequently conducted with three additional participants whose data were not included in the original datasets. The previously trained models were evaluated without retraining, fine-tuning, or participant-specific adaptation. The protocol for this additional evaluation is described in Section 2.7, and the corresponding results are presented in Section 3.7.

### 2.5. Comparative Classifier Evaluation and Repeated Experiments

To complement the primary recognition experiments and provide additional evidence regarding the suitability of the selected neural-network architectures, a comparative evaluation was conducted using conventional machine-learning classifiers. Specifically, Support Vector Machine (SVM) and Random Forest (RF) models were considered as alternative baselines for both static and dynamic Mexican Sign Language (MSL) recognition. The purpose of these experiments was to determine whether the selected MLP and TimeDistributed–LSTM architectures provided competitive classification performance compared with conventional approaches, while also examining the consistency of the results across different random seeds.

The comparative experiments were conducted using the same datasets described in Section 2.4, comprising 8550 static samples distributed across 29 classes and 750 dynamic sequences distributed across 10 classes. All samples originated from the same participant and were represented using hand landmarks extracted with MediaPipe Hands. Consequently, these experiments were designed to assess classification performance and repeatability within the available dataset rather than generalization to previously unseen signers.

For static sign recognition, the MLP, SVM, and RF classifiers were evaluated using the same 126-dimensional normalized feature vectors. Each vector represented the three-dimensional coordinates of 21 hand landmarks for up to two hands. The use of an equivalent input representation allowed the three classifiers to be compared without introducing differences in the dimensionality of the static feature vectors. The MLP architecture corresponded to the model described in Section 2.3.2, whereas SVM and RF were included as conventional classification baselines.

For dynamic sign recognition, the TimeDistributed–LSTM model processed the complete temporal sequence of 30 consecutive frames, with 126 normalized landmark features per frame, resulting in an input matrix of 30 × 126. In contrast, the SVM and RF classifiers required a fixed-length feature representation. Therefore, four statistical descriptors were calculated for each of the 126 landmark features across the temporal sequence: mean, standard deviation, minimum, and maximum. The concatenation of these descriptors produced a 504-dimensional feature vector for each dynamic sample.

This representation enabled the conventional classifiers to incorporate summary information about the temporal variation in hand landmarks without explicitly processing the ordered sequence of frames. However, because the LSTM processed the complete temporal sequence while SVM and RF used statistical descriptors, the dynamic experiments compared different classification pipelines rather than isolating the contribution of the classifier architecture.

To examine the repeatability of the classification results, additional experiments were performed using three random seeds (0, 3, and 7). The evaluation considered classification accuracy, macro-averaged F1-score, classification errors, and mean inference time per sample. Statistical variability was summarized using the standard deviation of classification accuracy across random seeds and the reported cross-validation folds.

The comparative evaluation complemented the primary stratified random and sequential 80/20 partitioning protocols described in Section 2.4. The primary experiments characterized the performance of the selected MLP and TimeDistributed–LSTM models under two data-partitioning strategies, whereas the additional experiments provided a broader comparison with conventional classifiers and an assessment of variability across repeated evaluations.

The reported inference times correspond to the classification stage and should not be interpreted as complete system-response times, which additionally involve image acquisition, hand-landmark extraction, decision processing, communication, and robotic actuation. Furthermore, because all experiments were conducted using the same single-participant datasets, the results do not establish signer-independent generalization or robustness under unrestricted acquisition conditions.

### 2.6. Recognition-to-Actuation Mapping and Robotic Reproduction

The connection between sign recognition and robotic actuation is implemented through a deterministic key-based lookup rather than through a second machine-learning model. Once the classification model produces an accepted class label, the system uses this label as a key to retrieve the predefined robotic motion assigned to the corresponding sign. Figure 2 presents representative robotic configurations resulting from this recognition-to-actuation mapping.

The resulting sequence of joint-angle commands is transmitted from the host computer to the ATmega2560 through UART. The microcontroller interprets the received angle values and generates the corresponding control signals for the robotic arm servomotors. Thus, the robotic response is generated through a predefined motion sequence associated with the recognized symbolic class.

The relationship between sign recognition and robotic actuation is therefore symbolic rather than geometric. The proposed system does not estimate the user’s hand pose and continuously reproduce its joint configuration on the robotic arm. Instead, the recognized MSL class serves as a symbolic command that selects a predefined robotic choreography.

This recognition-to-actuation relationship is important for interpreting the proposed system as an HCI architecture. The robotic arm functions as a physical feedback mechanism that provides an observable response to the user’s recognized gesture. The system consequently establishes a recognition-to-actuation interaction loop in which the human gesture is visually sensed, computationally classified, and used to trigger a corresponding physical robotic response.

The predefined robotic configurations were specified according to the MSL signs included in the experimental vocabulary. Accordingly, the current implementation focuses on discrete sign recognition and symbolic robotic reproduction rather than continuous motion imitation. Extending the system toward participant-independent evaluation, continuous hand-motion tracking, and automatic generation of robotic trajectories represents a potential direction for future research.

### 2.7. Preliminary Signer-Independent Evaluation Protocol

To complement the within-subject evaluation, a preliminary signer-independent assessment was conducted with three additional participants who were not involved in the construction of the training or evaluation datasets.

The previously trained MLP and TimeDistributed–LSTM models were evaluated without retraining, fine-tuning, or participant-specific adaptation. Therefore, no samples from these additional participants were incorporated into model training.

The evaluation included 39 sign classes, comprising the 29 static and 10 dynamic classes considered in the original recognition experiments. Each participant performed five recognition attempts per class, resulting in 195 attempts per participant and 585 attempts overall.

A recognition attempt was considered correct when the class predicted by the system matched the sign requested from the participant. Recognition performance was calculated as the proportion of correct predictions relative to the total number of attempts.

This additional experiment was designed to provide preliminary evidence of recognition performance with previously unseen signers rather than to establish comprehensive generalization across the broader Mexican Sign Language user population.

## 3. Results

### 3.1. Dataset and Experimental Evaluation

The dataset used to evaluate the proposed vision-based HCI system was collected using the same RGB camera for both static- and dynamic-sign acquisition. The experimental datasets incorporated controlled variations in acquisition conditions. For the static dataset, variation was introduced through hand position and camera distance, whereas the dynamic dataset additionally incorporated variations in camera viewpoint, background, clothing, and visual elements such as caps and eyeglasses.

For the static recognition branch, 29 classes were considered, comprising 21 letters of the MSL manual alphabet and 8 numerical signs. For 28 classes, 300 samples were collected, whereas 150 samples were available for the letter T. Therefore, the static dataset comprised a total of 8550 samples. Each sample was represented by 126 normalized landmark values extracted from the acquired image. To introduce spatial variation during acquisition, the signs were performed at three positions within the camera frame (right, center, and left) and at two distances from the camera (near and far).

For the dynamic recognition branch, 10 classes were considered, comprising 6 letters, 2 numerical signs, and the words “Hola” and “Adiós”. Each class contained 75 temporal sequences, resulting in a total of 750 temporal sequences. Each sequence consisted of 30 consecutive frames acquired at 30 frames per second (fps), corresponding to a temporal span of approximately 1 s. Each sequence corresponded to a separate acquisition of a complete sign execution rather than to an overlapping temporal window extracted from a continuous recording. The acquisitions included variations in camera viewpoint, background, clothing, and additional visual elements such as caps and eyeglasses. Each sequence was represented by a (30 × 126) feature matrix after hand-landmark extraction and normalization.

For the primary evaluation, an 80/20 stratified random partition was used for training and evaluation, preserving the class proportions in both subsets. Consequently, the static model was evaluated using 1710 evaluation samples, whereas the dynamic model was evaluated using 150 evaluation sequences.

To examine the sensitivity of recognition performance to the data-partitioning strategy, a second sequential protocol was subsequently applied. In this protocol, complete samples were ordered according to their acquisition sequence, with the first 80% used for training and the final 20% reserved exclusively for evaluation. For the dynamic dataset, a complete 30-frame sequence was treated as a single sample during this process; therefore, no frames from an individual sequence could appear in both subsets. A new instance of each classification model was trained using the same model configuration and training criteria described in Section 2.3.

The resulting models were then evaluated exclusively using the previously unseen evaluation set.

The performance of both recognition branches was evaluated using accuracy, precision, recall, F1 score, and confusion matrices.

### 3.2. Static Sign Recognition

The static recognition branch achieved perfect classification performance under the stratified random partitioning protocol. The model correctly classified all 1710 evaluation samples corresponding to the 29 static classes, resulting in an accuracy of 100.00%. Precision, recall, and F1-score were equal to 1.000 for all classes. The corresponding confusion matrix is presented in Figure 3, where all observations are located along the main diagonal and no misclassified samples are observed.

Under the sequential partitioning protocol, the static model achieved an accuracy of 99.88% on the 1710 held-out evaluation samples. Only two classification errors were observed: one sample from class “O” was incorrectly classified as “V”, and another sample from class “O” was incorrectly classified as “F”. These two errors represent the only deviations from correct classification across the 29 classes.

The corresponding confusion matrix is shown in Figure 4. The matrix is predominantly diagonal, with two off-diagonal elements corresponding to the misclassifications from class “O” to classes “V” and “F”. The F1 score was 0.983 for class “O”, whereas the F1 scores for classes “V” and “F” were both 0.992. All remaining classes achieved an F1 score of 1.000.

The accuracy decreased from 100.00% under the random partitioning protocol to 99.88% under the sequential protocol, corresponding to two misclassified samples among the 1710 evaluation samples in the latter case.

### 3.3. Dynamic Sign Recognition

The dynamic recognition branch achieved 100.00% accuracy under the stratified random partitioning protocol. All 150 evaluation sequences belonging to the 10 dynamic classes were correctly classified, resulting in precision, recall, and F1 scores of 1.000 for all classes. The corresponding confusion matrix is presented in Figure 5 and shows a fully diagonal distribution, with no misclassified sequences.

The dynamic model also achieved 100.00% accuracy under the sequential partitioning protocol. No classification errors were observed among the 150 sequences reserved for the evaluation set. Precision, recall, and F1 score remained equal to 1.000 across all 10 dynamic classes.

As shown in Figure 6, all sequences are located along the main diagonal, indicating that none of the dynamic classes was confused with another under the sequential evaluation protocol.

The results indicate that the temporal classification branch maintained its performance when evaluated on sequences acquired after the training block. In contrast to the static branch, no decrease in accuracy was observed between the random and sequential evaluation protocols.

### 3.4. Comparative Analysis of Recognition Performance

The results obtained by the two recognition branches under the two partitioning protocols are summarized in Table 1.

Overall, both recognition branches exhibited high classification performance across the two evaluation protocols. The static branch showed a small reduction in accuracy under sequential partitioning, from 100.00% to 99.88%, whereas the dynamic branch maintained an accuracy of 100.00% under both protocols.

The confusion matrices shown in Figure 3, Figure 4, Figure 5 and Figure 6 further illustrate the classification behavior. Three of the four matrices are completely diagonal, whereas the only observable deviations occur in the static model under sequential partitioning. These deviations involve classes “O”, “V”, and “F”, with both misclassifications originating from class “O” and being incorrectly assigned to classes “V” and “F”.

### 3.5. Recognition-to-Robotic Response

Following successful classification, the recognized class was used as the input to the deterministic recognition-to-actuation mapping described in Section 2.6. Each class label was associated with a predefined sequence of joint-angle configurations, which was transmitted to the ATmega2560 through the UART interface.

The physical robotic response was evaluated in 92 independent sign-recognition trials. In all 92 trials, each accepted prediction generated the corresponding UART command and triggered the associated predefined robotic sequence, yielding a 100% observed robotic actuation success rate during the end-to-end evaluation.

The joint-angle commands were defined according to the physical assembly of the robotic prototype. Because the servos were mounted in different orientations, including upside-down configurations, the positive direction of motion was established individually for each actuator. Consequently, different angular commands could produce motion in the same intended direction of the robotic arm. Rather than using a universal zero-angle reference, an initial set of servo angles was experimentally defined to represent the resting position of the assembled prototype. All subsequent configurations used this posture as their reference. The commanded servo angles were restricted to 10–170° to avoid operation near the nominal angular endpoints of 0° and 180°.

The robotic response depended directly on the discrete classification output: a correct prediction selected the predefined choreography associated with the intended class, whereas an incorrect prediction would trigger the choreography associated with the predicted class. Thus, the system performed deterministic recognition-to-actuation mapping rather than continuous kinematic imitation of the user’s hand movements.

To quantitatively evaluate the angular positioning accuracy, repeatability, and command-execution reliability of the robotic subsystem, an additional experiment was conducted independently of the sign-recognition models. In this experiment, 92 predefined joint-angle configurations were tested, with each configuration repeated 10 times, resulting in a total of 920 command-transmission attempts. The commands were sent directly from the host computer to the ATmega2560 through the UART interface, allowing the robotic actuation subsystem to be evaluated without involving the sign-recognition stage. The actual joint angles were measured using a camera and verified with a protractor.

Of the 920 command-transmission attempts, 911 resulted in successful robotic actuation, whereas nine did not produce movement because the corresponding UART commands were not received. These results correspond to an observed command-execution success rate of 99.02% and a failure rate of 0.98%. The nine unsuccessful attempts were excluded from the angular positioning analysis because the commanded movements were not executed.

Angular positioning accuracy was evaluated by comparing the commanded and measured joint angles for the 911 successful executions. The mean absolute percentage error (MAPE) was 2.999%, while the mean absolute angular error (MAE) was 2.574°. Repeatability was assessed by calculating the sample standard deviation of the measured joint angles across the valid repetitions of each commanded configuration. The mean within-configuration standard deviation was 3.143°. These results provide a quantitative characterization of the positioning accuracy and repeatability of the assembled robotic prototype under the evaluated conditions, rather than the nominal positioning accuracy of individual servomotors.

The temporal response was characterized in terms of processing rate and recognition-to-command latency. During continuous operation, 2789 frames were processed at an average rate of 23.09 frames per second (FPS), corresponding to 43.31 ms per iteration. This measurement included image acquisition, MediaPipe hand-landmark extraction, model inference when applicable, and HUD updates. For the 92 accepted sign predictions that generated UART commands, the mean latency between prediction acceptance and command transmission was 58.93 ms. A prediction was considered accepted when its confidence exceeded the predefined threshold of 0.85 and the cooldown condition was satisfied. This latency therefore represents the measured recognition-to-command transmission time, not the complete human-gesture-to-robot-motion latency.

Because the servos do not provide position feedback to the microcontroller, the physical completion time of each movement could not be measured directly. Instead, actuation time was estimated from the manufacturer-specified speed of the slowest servo (0.14 s/60° at 6 V). For a reference displacement of 160°, the estimated actuation time was 373.33 ms. Adding this estimate to the measured recognition-to-UART latency yielded an estimated recognition-to-actuation time of

*T_recognition_*_−_*_to_*_−_*_actuation_* = *T_recognition_*_−_*_to_*_−_*_UART_* + *T_actuation_*

*T_recognition_*_−_*_to_*_−_*_actuation_* = 58.93 ms + 373.33 ms

*T_recognition_*_−_*_to_*_−_*_actuation_* = 432.26 ms

This value is an estimate for the reference displacement, not a direct measurement of the complete human-gesture-to-robot-motion latency because neither gesture onset nor physical movement completion was independently instrumented.

Overall, the results characterize the functional integration and temporal behavior of the proposed MSL-to-robot interaction pipeline. The independent robotic evaluation additionally provides quantitative evidence of angular positioning accuracy, repeatability, and observed command-execution reliability. The 100% success rate obtained in the 92 end-to-end trials refers to the activation of the predefined robotic sequences, whereas the 99.02% success rate obtained in the 920 additional trials characterizes command execution under the independent evaluation conditions. Although the angular measurements quantify final positioning performance, they do not establish trajectory-tracking accuracy or continuous movement fidelity. Likewise, the recorded communication outcomes indicate whether the robot executed the transmitted commands, rather than providing a protocol-level assessment of UART data integrity. The contribution of the prototype therefore lies in integrating established techniques for landmark extraction, neural classification, temporal sequence modeling, and deterministic robotic actuation into a single interaction pipeline, rather than in proposing a novel recognition algorithm.

### 3.6. Comparative Classifier Performance and Statistical Variability

Additional experiments were conducted to compare the recognition performance of the proposed MLP and TimeDistributed–LSTM architectures with conventional SVM and RF classifiers. The evaluation also considered repeated experiments using three random seeds (0, 3, and 7) to examine the variability of the classification results.

For static sign recognition, all three classifiers achieved a mean accuracy of approximately 99.99%, with a macro-averaged F1 score of 0.9999. The MLP exhibited a standard deviation of 0.01 percentage points across random seeds and 0.02 percentage points across validation folds. The SVM and RF classifiers exhibited similarly low variability, indicating comparable classification performance across the evaluated configurations. The mean inference times were 0.144 ms, 0.148 ms, and 0.089 ms per sample for MLP, SVM, and RF, respectively. These results are summarized in Table 2.

For dynamic sign recognition, the TimeDistributed–LSTM model achieved the highest mean accuracy (99.96%), followed by RF (99.86%) and SVM (98.93%). The corresponding macro-averaged F1-scores were 0.9996, 0.9986, and 0.9893, respectively. The LSTM exhibited a standard deviation of 0.07 percentage points across random seeds and 0.09 percentage points across validation folds. In comparison, RF exhibited standard deviations of 0.11 and 0.21 percentage points, whereas SVM exhibited standard deviations of 0.20 and 0.67 percentage points, respectively.

Regarding computational performance, the mean inference times for the dynamic models were 6.006 ms for LSTM, 0.568 ms for RF, and 0.061 ms for SVM. The results indicate that LSTM achieved the highest mean classification accuracy and lowest variability among the evaluated dynamic recognition pipelines, although at a higher computational cost. The comparative results are presented in Table 3.

The comparative experiments provide complementary information to the primary recognition results presented in Section 3.2 and Section 3.3. While the original evaluation focused on the selected MLP and TimeDistributed–LSTM architectures under random and sequential data-partitioning protocols, the additional experiments examined the performance of alternative classifiers and the variability associated with repeated evaluations. The results indicate that the normalized hand-landmark representation supports high classification accuracy across different models for static signs. For dynamic recognition, the TimeDistributed–LSTM pipeline achieved the highest mean accuracy among the evaluated approaches, although with greater inference time. These findings support the suitability of the selected architectures for the present proof-of-concept implementation without establishing their general superiority or signer-independent generalization.

### 3.7. Preliminary Signer-Independent Recognition Results

A preliminary evaluation was conducted with three additional participants whose data were not included in the original datasets. The previously trained recognition models were used without retraining or participant-specific adaptation.

Across 585 recognition attempts involving 39 static and dynamic sign classes, 521 predictions were correct, corresponding to an overall recognition accuracy of 89.06%.

Participant 1 achieved 89.23% accuracy (174/195), Participant 2 achieved 90.26% (176/195), and Participant 3 achieved 87.69% (171/195).

These results provide preliminary evidence that the proposed recognition system can identify signs performed by individuals who were not represented in the training dataset. Nevertheless, the evaluation involved only three additional participants and a limited number of repetitions per class. Consequently, the findings should be interpreted as an initial assessment of signer-independent performance rather than definitive evidence of generalization to the broader MSL user population.

## 4. Discussion

### 4.1. Recognition Performance and Robustness

The experimental results indicate that the proposed architecture achieved high recognition performance for the selected static and dynamic MSL classes under the evaluated acquisition conditions. However, since all samples used in the original training and evaluation datasets were obtained from a single participant, these findings characterize within-subject performance rather than signer-independent generalization. The difference between the static branch’s random and sequential evaluation results was minimal, suggesting that its performance remained largely consistent across the evaluated data partitions. Nevertheless, these within-subject results do not establish complete robustness to variations in acquisition conditions or demonstrate generalization to other signers.

The preliminary signer-independent evaluation provides complementary evidence regarding the recognition system’s ability to process signs performed by previously unseen participants. Unlike the original within-subject experiments, this assessment involved three additional signers whose data were excluded from model training and whose recognition attempts were evaluated without model adaptation. The overall accuracy of 89.06% was lower than the near-perfect accuracies obtained with the original single-participant dataset, suggesting that inter-signer differences may affect recognition performance. Although these results provide preliminary evidence of recognition beyond the training participant, the limited number of additional signers prevents definitive conclusions regarding robustness and generalization to the broader MSL population.

The errors observed in the static branch highlight a potential limitation of relying exclusively on hand-landmark geometry to distinguish signs with similar configurations. Although three-dimensional landmarks provide spatial information beyond that available from conventional two-dimensional image representations, recognition may still be affected by subtle differences in finger articulation, hand orientation, landmark localization, and partial occlusion. These factors may be particularly relevant when distinguishing visually similar hand configurations. The observed errors therefore point to opportunities for improving the robustness of the static representation rather than indicating a fundamental limitation of the overall recognition approach.

In contrast, the dynamic branch maintained consistent performance across the two evaluation protocols. For the selected vocabulary and the evaluated signer, this behavior suggests that temporal information provided additional discriminative cues. The combination of frame-level feature processing and stacked LSTM layers enables the model to represent both the spatial configuration of the hands and its evolution over time. However, the absence of observed classification errors should be interpreted within the scope of the experimental dataset and not as evidence of unrestricted recognition performance. Evaluation with multiple signers, subject-independent data partitions, and less controlled acquisition conditions will be necessary to determine the extent to which these findings generalize to broader MSL recognition scenarios.

The additional experiments involving different random seeds provide further evidence regarding the repeatability of the recognition results. Both neural-network architectures exhibited limited performance variability across the evaluated configurations, with standard deviations across seeds of 0.01 and 0.07 percentage points for static and dynamic recognition, respectively. These findings indicate that the high classification accuracies were not restricted to a single random seed. Nevertheless, the repeated experiments were conducted using the same single-participant datasets, and therefore, the observed stability should be interpreted as within-dataset repeatability rather than evidence of generalization across different signers or acquisition environments.

### 4.2. Comparison with Existing Sign Language Recognition Approaches

The comparison with alternative classifiers provides additional insight into the selection of the recognition architectures. For static signs, the MLP, SVM, and RF classifiers achieved nearly identical mean accuracies, suggesting that the normalized hand-landmark representation was sufficiently discriminative for the evaluated vocabulary regardless of the selected classifier. Consequently, the experimental results do not establish a substantial classification advantage for the MLP over conventional machine-learning methods.

For dynamic signs, the TimeDistributed–LSTM architecture achieved the highest mean accuracy and lowest variability among the evaluated pipelines. This result supports its suitability for processing the temporal sequences considered in this study. However, the SVM and RF classifiers used statistical summaries of the temporal features rather than the complete frame sequences, and therefore, the comparison does not isolate the contribution of the recurrent architecture from differences in input representation. Furthermore, the shorter inference times obtained with the conventional classifiers indicate a trade-off between classification performance and computational efficiency. These findings support the selection of the LSTM for the present proof-of-concept implementation while highlighting the relevance of further comparisons involving alternative temporal architectures.

The proposed system occupies an intermediate position between wearable sensing approaches and camera-based computer-vision approaches reported in the literature. Instrumented gloves, such as those described in [11,12], directly measure hand or finger movements through physical sensors. These systems can provide direct measurements of hand-related physical variables and are less dependent on illumination, background, or camera framing. However, they require the user to wear additional hardware, introducing an additional instrumentation requirement during interaction. In contrast, the proposed approach uses a conventional RGB camera and contactless hand-landmark extraction, eliminating the need for instrumented gloves or other wearable sensors.

The comparison with [11,12] therefore highlights a fundamental design trade-off. Wearable systems provide direct sensing of physical variables but require user instrumentation, whereas the proposed system favors contactless interaction and simpler hardware requirements at the cost of greater dependence on visual acquisition conditions. This trade-off is particularly relevant to HCI applications because the sensing mechanism itself influences the interaction requirements. A camera-based interface can operate without requiring the user to attach or calibrate a physical device, which may be advantageous in shared or demonstration environments.

The proposed architecture is also consistent with the computer-vision paradigm based on hand-landmark extraction followed by machine-learning classification. Previous MediaPipe-based approaches [14,15] have demonstrated that compact hand representations can provide an effective alternative to directly processing full RGB images.

Mejía Pérez and Córdova Esparza [15] investigated Mexican Sign Language recognition using neural-network-based methods. Their study addressed recognition of MSL signs using a different experimental configuration and recognition methodology. In contrast, the present study uses an RGB camera and MediaPipe Hands to extract wrist-relative hand landmarks, with separate MLP and LSTM classifiers for static and dynamic signs, respectively. Furthermore, the proposed system extends recognition to deterministic robotic reproduction through a host-computer and ATmega2560 architecture. These differences in sensing modality, input representation, class composition, and system objectives mean that the reported performance values should not be interpreted as a direct benchmark between the two approaches. Instead, the studies represent complementary approaches to MSL recognition, with the present work emphasizing the integration of visual recognition and robotic actuation.

The present work adopts a related approach but explicitly distinguishes between static and dynamic signs. Static signs are processed using an MLP operating on a single 126-value landmark vector, whereas dynamic signs are represented as temporal sequences of 30 frames and classified using a TimeDistributed–LSTM architecture. This separation is relevant because static and dynamic signs contain different types of discriminative information. A static classifier does not need to model temporal dependencies, whereas dynamic recognition must account not only for the spatial configuration of the hands but also for their temporal evolution.

The use of a flat landmark vector can also be considered in the broader context of structure-aware recognition. Explicit modeling of object components and their relationships predates graph neural networks, with early examples including hierarchical parsing and recognition of hand-sketched diagrams [22] and multi-layer parsing strategies for hand-drawn diagrams [23]. More recent sign-language recognition approaches have incorporated this principle through skeleton-based representations that preserve spatial relationships among joints [24], including tree-structured skeleton representations evaluated on Mexican Sign Language data [25]. Graph convolutional approaches have further formalized this structural representation by modeling skeleton joints as graph nodes and their physical relationships as graph edges [24]. More recent hand-aware graph-based methods model hand topology to capture local relationships among hand joints [26].

In contrast, the proposed system represents the two hands as a flat 126-value vector derived from 21 three-dimensional landmarks per hand. The representation therefore does not explicitly encode connectivity or anatomical relationships among joints. The main advantage of this formulation is its compactness and computational simplicity, allowing the landmark coordinates to be directly processed by conventional MLP and LSTM architectures without requiring an explicit graph-construction stage or graph-based message-passing operations. This formulation also simplifies feature preparation and facilitates integration of the recognition models into the proposed interaction pipeline. However, simplified representation may discard structural information that could be useful for distinguishing signs with similar hand configurations. Relationships such as the connectivity between adjacent joints and the hierarchical organization of the fingers are not explicitly provided to the classifier and must instead be inferred from the coordinate values. Therefore, the choice of flat representation constitutes a deliberate trade-off between representational simplicity and explicit structural modelling.

This methodological difference is also relevant when comparing the proposed system with recent MediaPipe-based approaches for MSL recognition. Rodriguez et al. [27] used MediaPipe-based hand landmarks for MSL recognition and evaluated 29 dactylological classes and the first ten numerical digits, while Sánchez-Vicinaiz et al. [28] combined MediaPipe-based feature extraction with convolutional neural networks for static MSL fingerspelling. These studies demonstrate the applicability of MediaPipe-derived representations to MSL recognition while adopting different classification strategies. The present work follows the same general landmark-based sensing paradigm but combines a flat 126-value representation with separate MLP and LSTM models and integrates the recognition output with a physical robotic response. Consequently, the comparison is not limited to classification performance but also concerns the representation and interaction architecture used to transform visual information into a robotic response.

This design also differs from more complex approaches based on hybrid spatial-temporal architectures. Studies using attention mechanisms, LSTM networks, or combinations of LSTM and three-dimensional convolutional networks [11,12,13] have reported high recognition performance, including values approaching or exceeding 99% under specific experimental conditions. Such architecture can provide greater representational capacity, particularly for larger vocabulary and more complex temporal patterns. However, their computational and implementation requirements may also be greater. The proposed architecture instead uses hand landmarks as a compact intermediate representation, reducing the dimensionality of the input before classification. This design favors architectural simplicity and provides a potential basis for future deployment on edge-computing platforms.

The comparison with embedded sign-language recognition systems [11,14] is also relevant. Unlike fully embedded approaches, the current implementation performs image acquisition, landmark extraction, and neural-network inference on a host computer, while the ATmega2560 is dedicated to robotic actuation. Therefore, the proposed system should not be considered a fully embedded vision system. Its contribution instead lies in the integration of a vision-based recognition module with a physical robotic response. The relatively compact input representation and classification architecture nevertheless make the recognition stage a potential candidate for future implementation on more capable edge-computing hardware.

The comparison with Mexican Sign Language research is particularly relevant. Previous studies on Mexican Sign Language (MSL) [15,16] have explored recognition using multimodal information obtained from RGB-D or depth-based systems, including hand, body, and facial information. These approaches provide a richer description of the signer and can therefore represent linguistic elements that cannot be inferred from isolated hand geometry alone. The present system adopts a more restricted representation based exclusively on hand landmarks obtained from a conventional RGB camera. This results in a simpler sensing configuration but also establishes an explicit limitation: the current system is primarily designed for isolated signs in which hand configuration and/or hand movement provide sufficient discriminative information.

This distinction is particularly relevant because MSL, as a natural language, involves more than manual hand configurations. Facial expressions, body posture, head movement, spatial relationships, and non-manual grammatical markers can contribute to meaning. Consequently, the proposed system should be understood as an MSL sign-recognition platform rather than as a complete MSL translation system. The studies in [15,16] provide a relevant reference for future expansion toward multimodal recognition through the incorporation of body and facial information.

An additional distinction emerges when the proposed system is compared with robotic sign-generation approaches [19,20,21]. These studies demonstrate the feasibility of using robotic hands or manipulators to reproduce sign-language configurations. The present work follows a related principle in which the recognized class is associated with a predefined robotic sequence. However, the contribution is not limited to robotic sign generation; rather, the robotic actuator is incorporated as the output stage of a complete vision-based HCI pipeline.

This interaction chain distinguishes the proposed architecture from systems that address recognition and robotic reproduction independently. Importantly, the robot does not continuously imitate the user’s hand kinematics. Instead, it reproduces a predefined choreography associated with the recognized class. The distinction between gesture classification and kinematic imitation is therefore important for avoiding claims regarding capabilities that were not experimentally evaluated.

### 4.3. Human–Computer Interaction Perspective

Although the proposed system establishes a functional interaction pathway from visual gesture acquisition to robotic actuation, the evaluation primarily addresses technical performance. It should therefore be regarded as an HCI-oriented prototype rather than a fully validated interactive system. The robotic response provides an observable physical representation of the recognized sign, which may be relevant in educational or demonstrative settings. However, no user-centered evaluation was conducted to assess usability, accessibility, user acceptance, task completion, interaction performance, or educational benefits. These aspects require further investigation with representative end users.

The manual selection between static and dynamic recognition modes is another design consideration. Rather than automatically determining the temporal nature of an incoming gesture, the current implementation requires the user to select the appropriate recognition branch. This simplifies the processing pipeline but introduces an additional interaction step that may limit usability in applications requiring fully autonomous operation.

The system also incorporates a confidence threshold and a cooldown mechanism to regulate robotic actuation. These measures provide basic safeguards against low-confidence predictions and repeated activations in rapid succession. However, their effectiveness in preventing unintended responses and supporting satisfactory interaction has not been established through systematic user-centered evaluation. Future studies should therefore assess these parameters alongside usability and interaction performance.

### 4.4. Potential Relevance to Sustainable Development Goals 4 and 10

The proposed vision-based HCI system may contribute to future applications aligned with Sustainable Development Goal 4 (SDG 4: Quality Education) and Sustainable Development Goal 10 (SDG 10: Reduced Inequalities). By integrating contactless Mexican Sign Language (MSL) recognition with predefined robotic responses, the prototype provides a technological foundation that could be explored in interactive educational and accessibility-oriented environments. However, its potential contribution should be understood as complementary to existing educational resources and human instruction rather than as a demonstrated educational or social benefit.

The present study establishes technical feasibility but does not evaluate learning outcomes, usability, accessibility, or interaction with the intended Deaf community. Furthermore, although a preliminary evaluation with three additional signers was conducted, the limited number of participants prevents broader conclusions regarding generalization across the MSL user population. Therefore, the relationship with SDGs 4 and 10 remains prospective. Future research should include multi-participant validation and user-centered evaluations involving MSL users and educational specialists to determine whether the proposed system provides measurable educational or accessibility benefits.

### 4.5. Limitations and Future Research

Several limitations should be considered when interpreting the reported results. First, the original static and dynamic datasets were collected from a single participant. Consequently, the near-perfect recognition accuracies obtained under the stratified random and sequential partitioning protocols primarily characterize within-subject performance and do not independently establish generalization across different signers. To partially address this limitation, a preliminary signer-independent evaluation was conducted with three additional participants who were not represented in the training datasets. Using the previously trained models without retraining or participant-specific adaptation, the system correctly recognized 521 of 585 attempts, corresponding to an overall accuracy of 89.06%. Although these findings provide initial evidence of recognition performance with previously unseen signers, the limited number of participants and repetitions per class prevents broader conclusions regarding generalization across the MSL user population. Future research should therefore include larger and more diverse participant cohorts, additional acquisition conditions, and systematic analyses of signer-dependent variability, including differences in hand morphology, signing style, execution speed, and hand orientation.

Second, the experiments were conducted under controlled acquisition conditions. Although variations in hand position, acquisition distance, background, and clothing were introduced, the system was not systematically evaluated under uncontrolled illumination, complex backgrounds, severe occlusions, partial hand visibility, or simultaneous interactions with other objects. These factors may affect hand-landmark detection and, consequently, classification performance.

Third, the current representation is restricted to hand landmarks. This representation is appropriate for the selected isolated signs but does not capture non-manual components of MSL. Future versions should investigate multimodal representations that combine hand, body, facial, and head landmarks, building on the direction of previous MSL studies [15,16].

Fourth, although additional comparisons with SVM and RF classifiers and repeated experiments using different random seeds were incorporated, a dedicated component-level ablation study was not performed. Consequently, the individual contributions of feature normalization, temporal representation, network depth, and other architectural components have not been isolated experimentally. Moreover, the repeated experiments were limited to three random seeds, restricting the scope of the statistical variability analysis. Future investigations should include systematic ablation experiments, additional random seeds, and comparisons with alternative temporal architectures under equivalent input representations and evaluation protocols.

Fifth, the robotic output is based on discrete predefined joint configurations rather than continuous kinematic imitation. Therefore, the present work does not quantify the similarity between the user’s hand trajectory and the robot’s movement. Future research could introduce a kinematic mapping layer capable of transforming hand or body landmarks into continuous robot joint trajectories, thereby enabling a more direct form of physical imitation.

Sixth, although the landmark-based representations motivate investigation of future edge deployment, inference is currently performed on the host computer. Such deployment would require systematic evaluation of computational load, memory consumption, inference latency, energy consumption, and recognition performance.

Migration toward an edge-computing platform could reduce the system’s dependence on a general-purpose computer and potentially facilitate more portable and autonomous implementations. However, the feasibility of such a transition would depend on the computational capabilities of the selected hardware platform and its ability to maintain reliable recognition and robotic interaction under the intended operating conditions.

Finally, the present study represents the initial technical phase of a broader research effort in which the primary objective was to develop, integrate, and evaluate the proposed system before conducting user-centered assessments. Accordingly, usability, accessibility, and educational effectiveness were not evaluated in this study. These aspects are planned to be addressed in a subsequent research phase, involving Deaf and hard-of-hearing users, MSL instructors, and educational personnel. The corresponding findings are intended to be presented in a separate scientific article. Such evaluations could provide evidence regarding usability, accessibility, interaction acceptance, and potential educational applications. These evaluations would help establish whether the demonstrated technical capabilities can support practical educational and accessibility-oriented applications.

Overall, the results support the technical feasibility of recognizing the selected isolated MSL signs and triggering predefined robotic responses under the evaluated conditions. The preliminary evaluation with three additional signers provides initial evidence of recognition beyond the original training participant, although broader generalization remains to be established. However, the present findings do not establish robustness across different users, performance under unconstrained environments, educational effectiveness, or broader accessibility impact. Accordingly, the results and conclusions should be interpreted within the scope of a technical proof-of-concept evaluation rather than as evidence of generalized MSL recognition or demonstrated educational impact. Further multi-participant, cross-condition, and user-centered evaluations are required to assess these aspects.

## 5. Conclusions

This work presented a vision-based Human–Computer Interaction (HCI) prototype for Mexican Sign Language (MSL) recognition and robotic reproduction, integrating RGB image acquisition, hand-landmark extraction, machine learning, and robotic actuation into a unified interaction pipeline. The static MLP achieved 100.00% accuracy under stratified random partitioning and 99.88% under sequential partitioning, while the dynamic TimeDistributed–LSTM model achieved 100.00% accuracy under both protocols. These findings support the technical feasibility of the proposed landmark-based recognition approach for the selected MSL signs under the controlled experimental conditions evaluated in this study.

A central contribution of the work is the integration of visual sign recognition with physical robotic feedback. Rather than limiting the interaction to classification or visual output, the recognized sign is mapped to a predefined robotic choreography, establishing a technical pathway from human gesture acquisition through computational recognition to physical robotic response. This architecture provides a contactless interaction modality without requiring wearable sensing devices and establishes a technological basis for future human–robot interaction applications involving sign language.

The proposed system may also be relevant to Sustainable Development Goal 4 (Quality Education) and Sustainable Development Goal 10 (Reduced Inequalities), particularly in contexts involving MSL practice, inclusive interaction, and accessibility-oriented technologies. However, these potential contributions were not directly evaluated in the present study, as no educational intervention, learning-gain analysis, or user-centered assessment with Deaf and hard-of-hearing users, MSL instructors, or educational personnel was conducted. Therefore, the potential relevance to these SDGs should be understood as a prospective application context rather than as evidence of demonstrated educational or social impact.

The current implementation is limited by the single-participant dataset, controlled acquisition conditions, hand-only representation, and host-based inference. Future work will focus on multi-participant evaluation, expanded MSL vocabularies, multimodal hand–body–face representations, edge deployment, and user-centered evaluations with the intended community. Future investigations should assess usability, accessibility, user acceptance, and potential educational applications with representative users and stakeholders. These evaluations could establish the practical applicability of the proposed system in educational and accessibility-oriented contexts. Overall, the proposed system supports the feasibility of combining computer vision and robotics for sign-language-based HCI and provides a technical foundation for future studies on inclusive and accessible interaction.

## Figures and Tables

**Figure 1:**
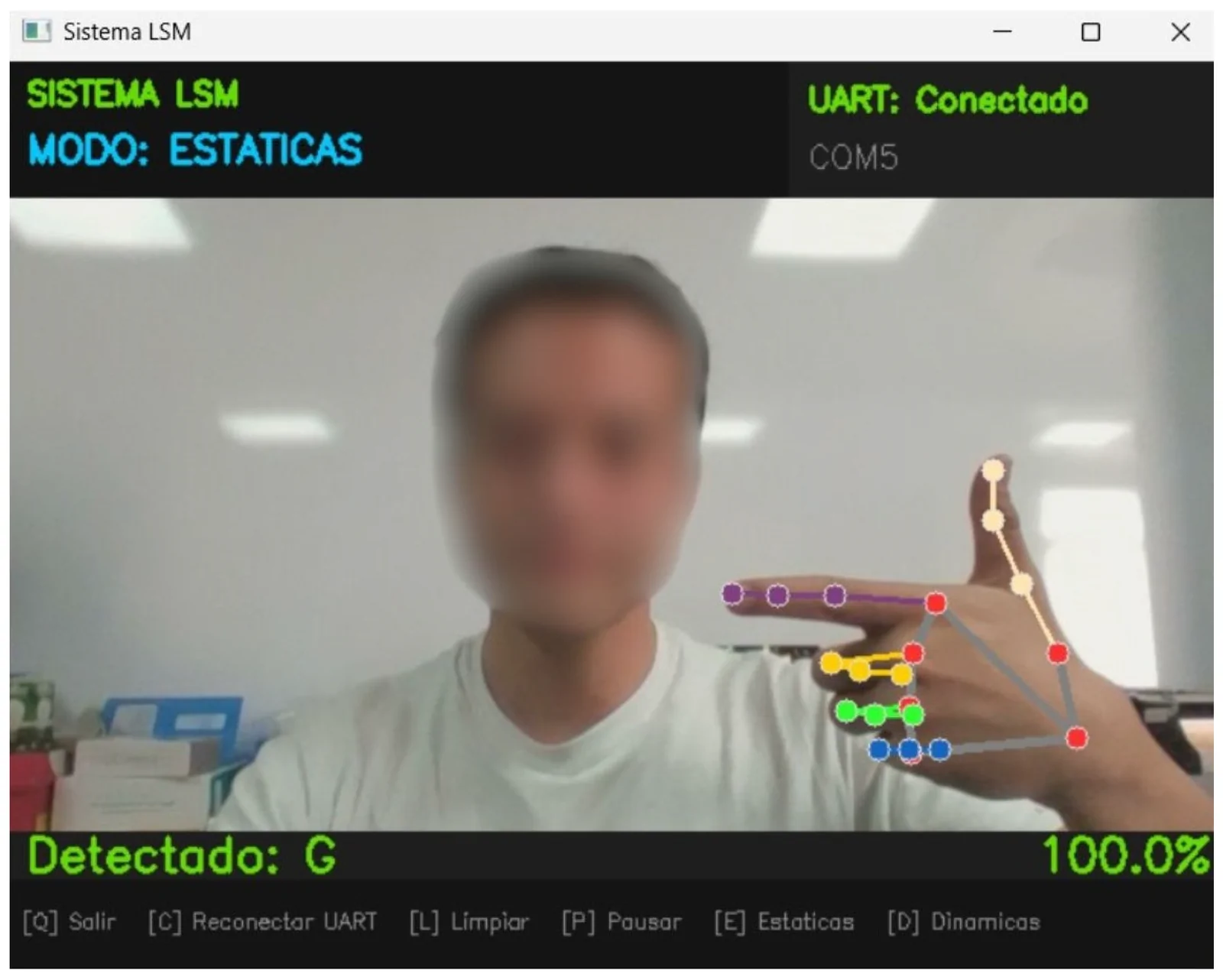
Vision-based HCI interface during system operation. The OpenCV-rendered HUD shows the active recognition mode, the most recently accepted gesture, classification confidence, UART communication status, and cooldown progress.

**Figure 2:**
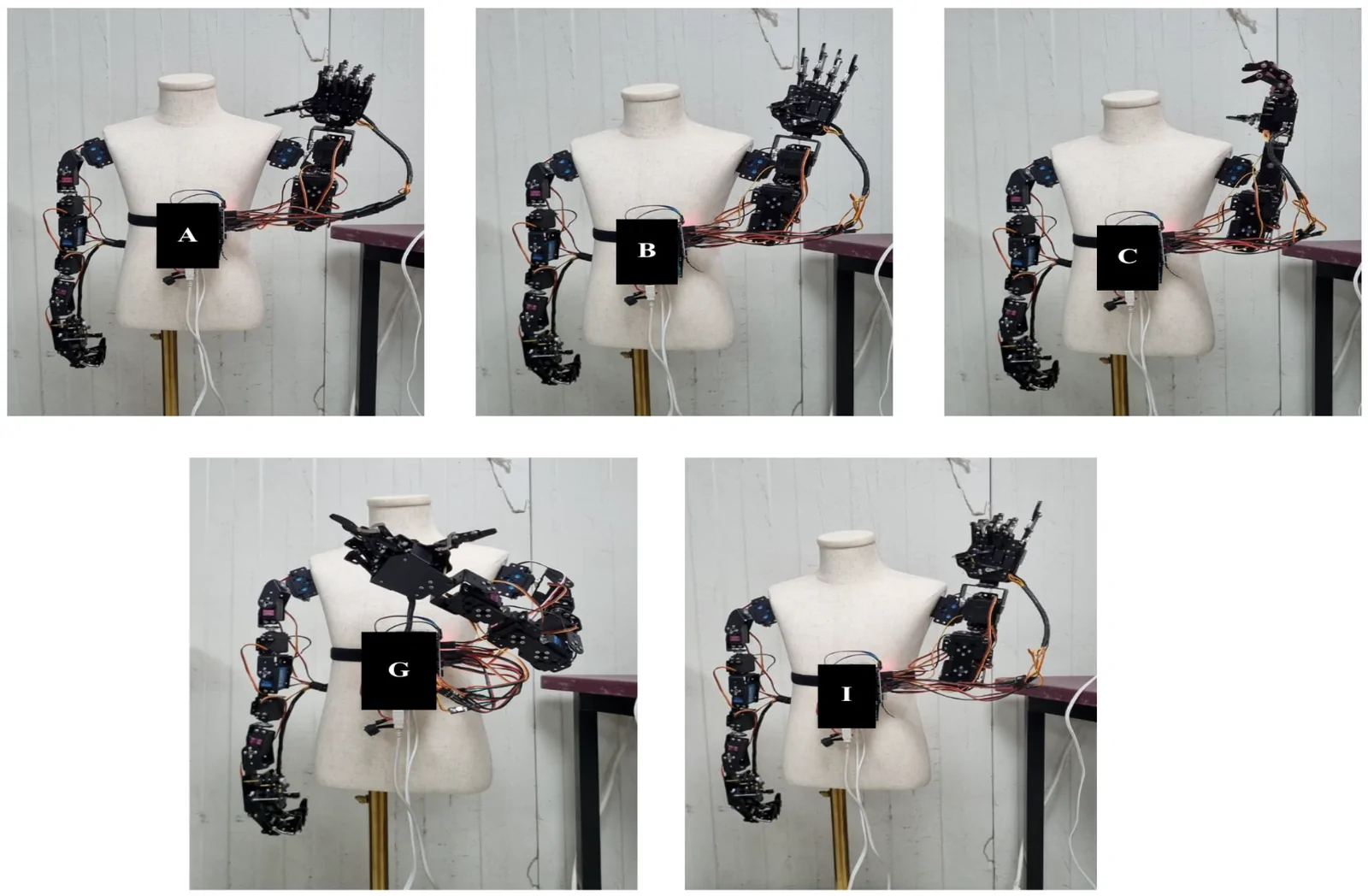
Robotic arm end-effector poses corresponding to a representative subset of MSL alphabet letters (A, B, C, G, and I). Each pose is retrieved through a deterministic mapping from the classification output to a predefined sequence of robotic joint configurations rather than being computed through inverse kinematics from the user’s hand geometry.

**Figure 3:**
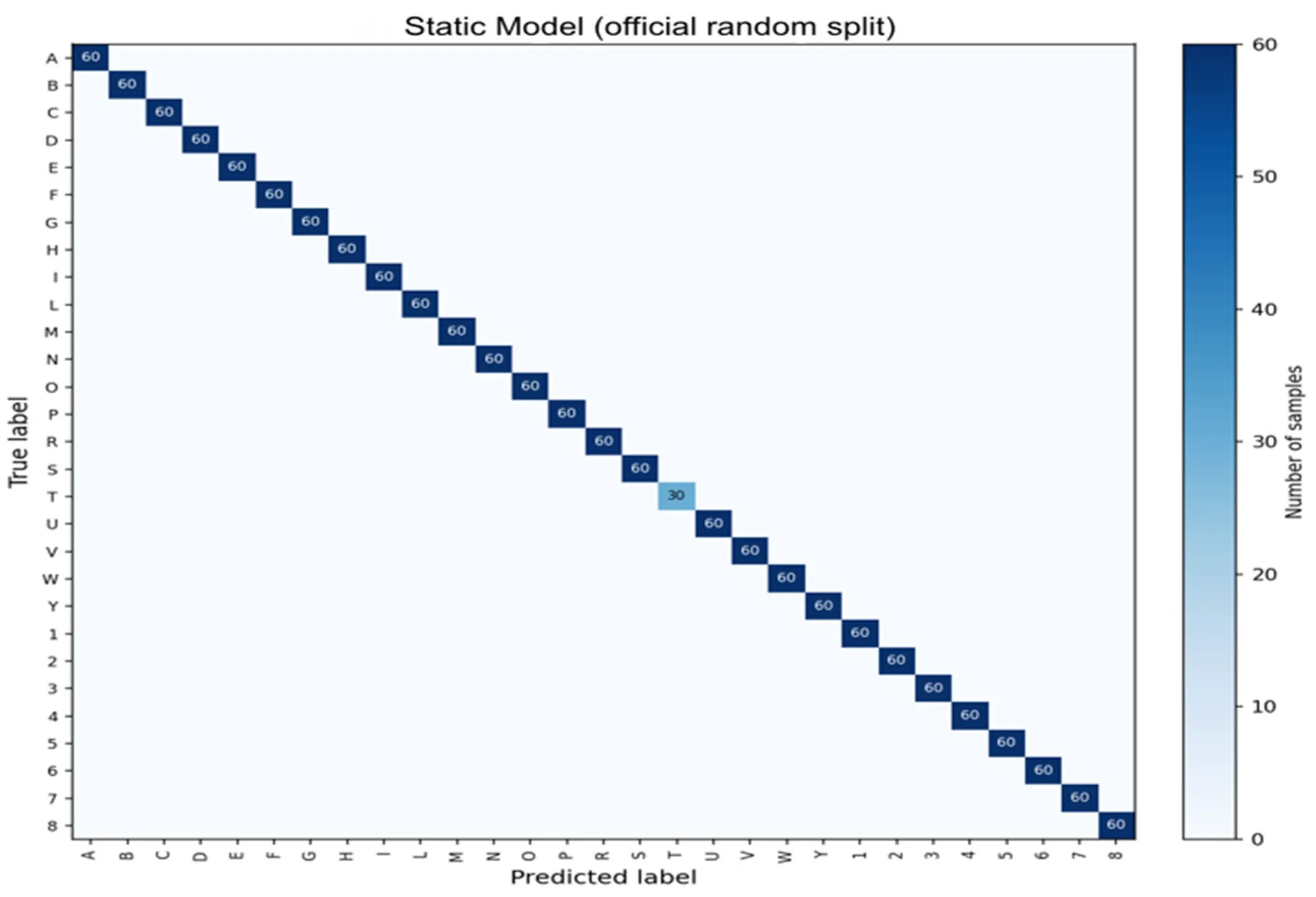
Confusion matrix of the static model under stratified random partitioning (1710 evaluation samples, 29 classes). All observations are located along the main diagonal, with no misclassified samples.

**Figure 4:**
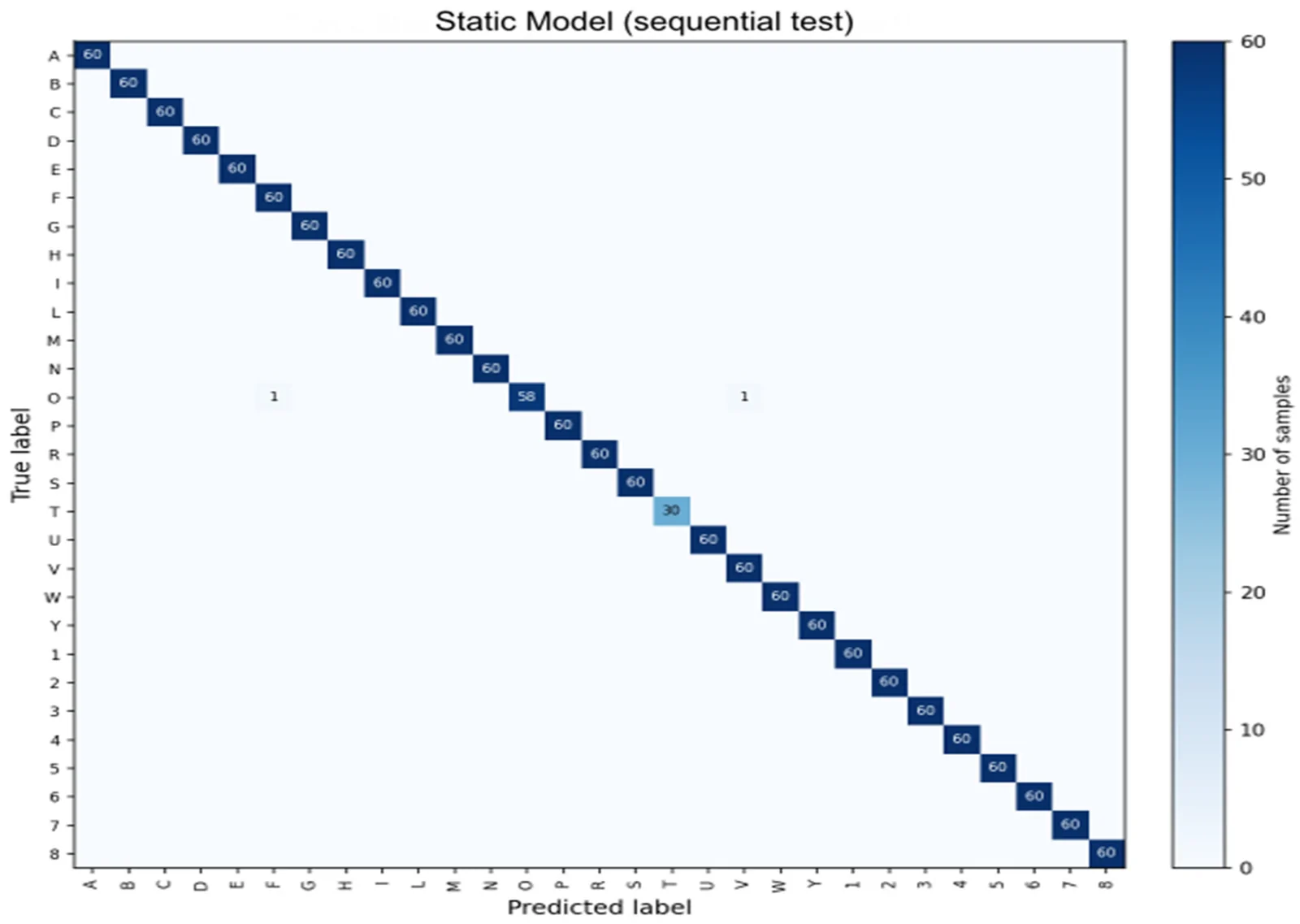
Confusion matrix of the static model under sequential partitioning (1710 previously unseen evaluation samples, 29 classes).

**Figure 5:**
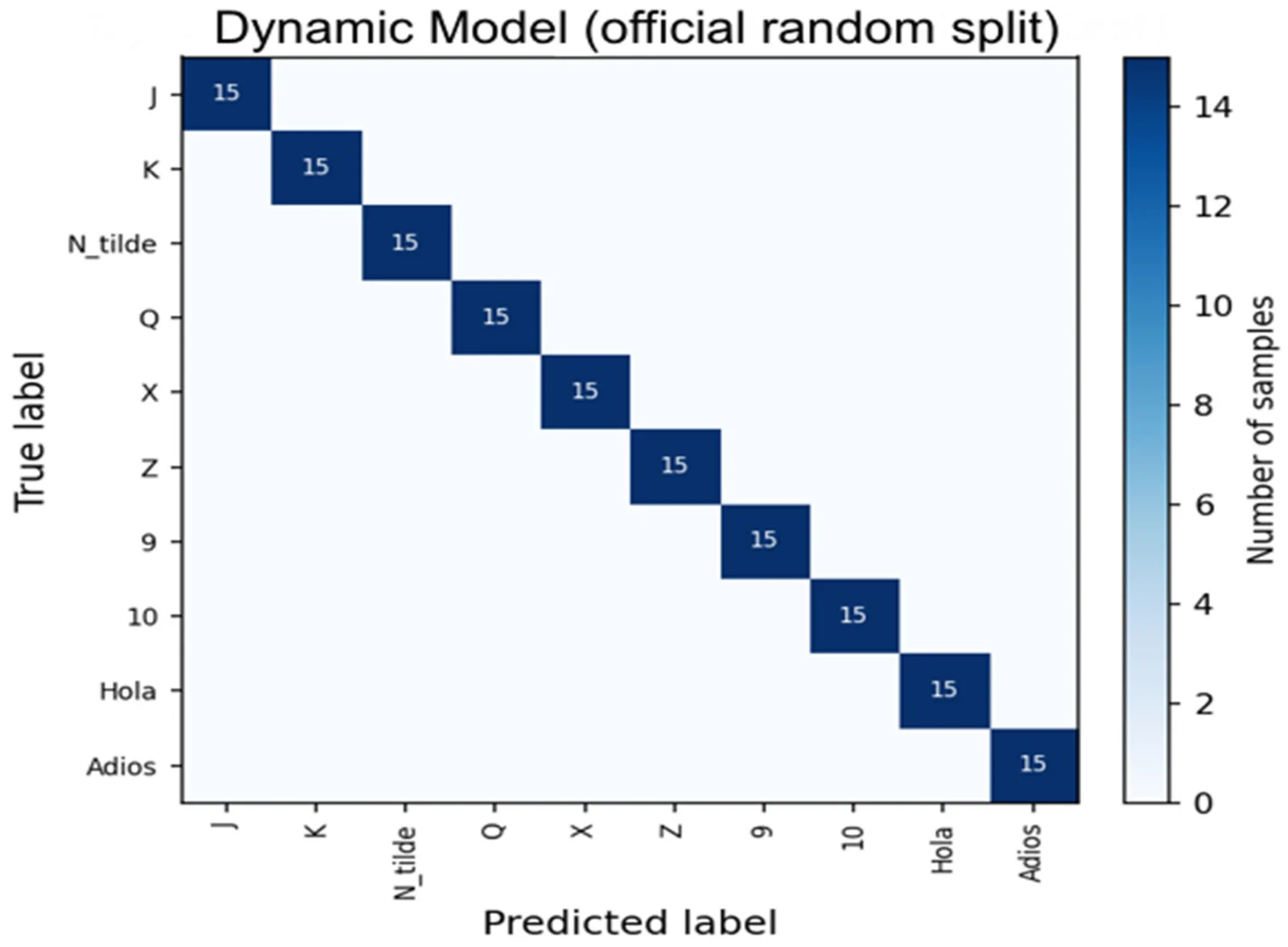
Confusion matrix of the dynamic model under stratified random partitioning (150 evaluation sequences, 10 classes).

**Figure 6:**
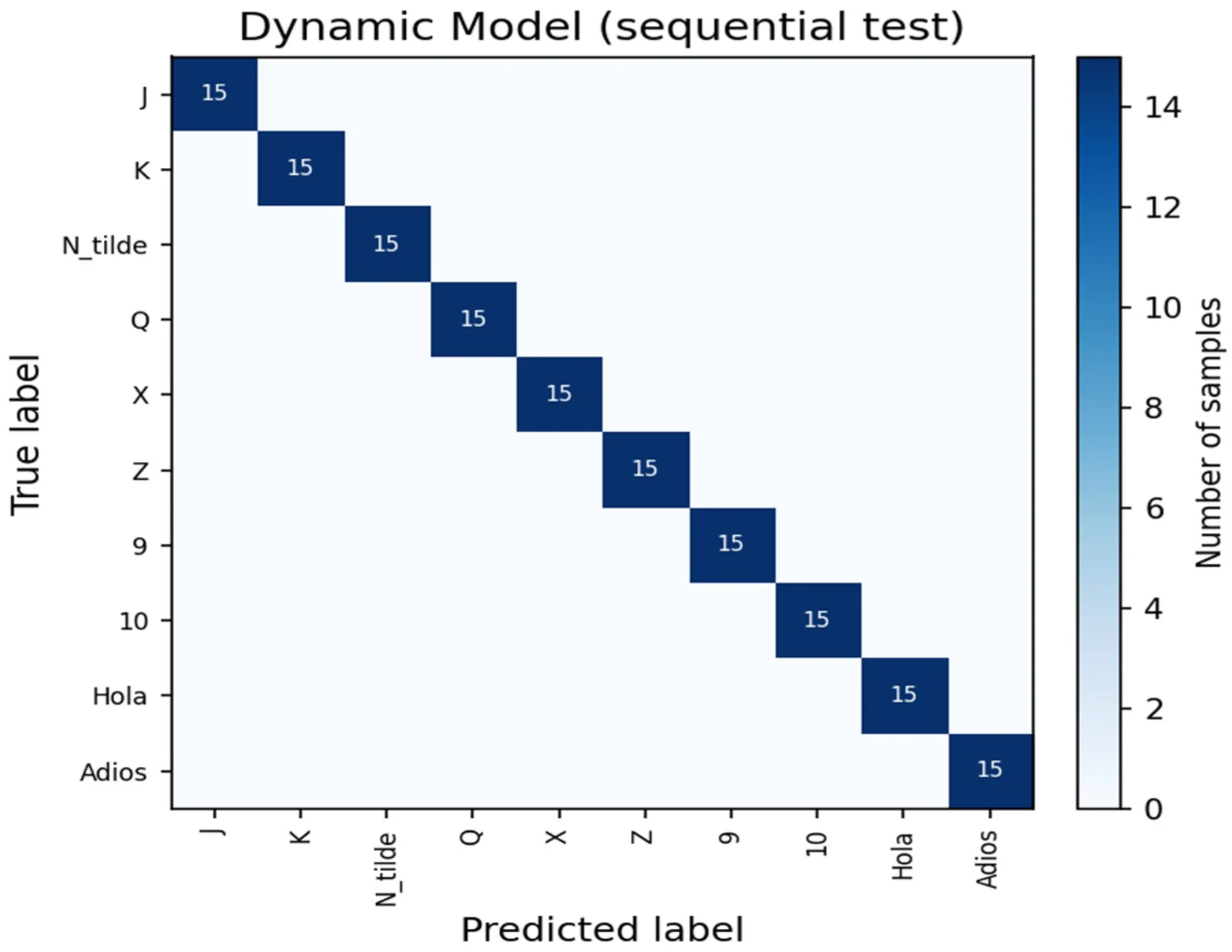
Confusion matrix of the dynamic model under sequential partitioning (150 previously unseen evaluation sequences, 10 classes).

**Table 1:** Recognition performance of the static and dynamic branches under the two data-partitioning protocols.

Recognition Branch	Partitioning Protocol	Evaluation Samples	Accuracy (%)	Precision	Recall	F1 Score
Static (MLP)	Stratified random	1710	100.00	1.000	1.000	1.000
Static (MLP)	Sequential	1710	99.88	0.999	0.999	0.999
Dynamic (TimeDistributed–LSTM)	Stratified random	150	100.00	1.000	1.000	1.000
Dynamic (TimeDistributed–LSTM)	Sequential	150	100.00	1.000	1.000	1.000

**Table 2:** Comparative classification performance and statistical variability of the evaluated models for static Mexican Sign Language recognition using three random seeds (0, 3, and 7).

Model	Mean Accuracy (%)	SD Seeds (pp)	SD Folds (pp)	Macro-F1	Mean Errors	Inference (ms)
MLP	99.99	0.01	0.02	0.9999	0.7	0.144
SVM	99.99	0.00	0.02	0.9999	1	0.148
Random Forest	99.99	0.00	0.02	0.9999	1	0.089

Note: All models used 126 normalized landmark features; dataset: 8550 samples, 29 classes. SD = standard deviation; pp = percentage points. Inference time is per sample.

**Table 3:** Comparative classification performance and statistical variability of the evaluated models for dynamic Mexican Sign Language recognition using three random seeds (0, 3, and 7).

Model	Mean Accuracy (%)	SD Seeds (pp)	SD Folds (pp)	Macro-F1	Mean Errors	Inference (ms)
TimeDistributed–LSTM	99.96	0.07	0.09	0.9996	0.3	6.006
SVM	98.93	0.20	0.67	0.9893	8	0.061
Random Forest	99.86	0.11	0.21	0.9986	1	0.568

Note: LSTM input: 30 × 126; SVM and RF input: 504 statistical features; dataset: 750 sequences, 10 classes. SD = standard deviation; pp = percentage points. Inference time is per sample.

## Data Availability

The datasets, trained models, and executable program developed for the proposed MSL recognition and robotic reproduction system are publicly available in the GitHub repository at https://github.com/JosueRiveraFernandez/Handspeak (accessed on 16 October 2026).

## Abbreviations

The following abbreviations are used in this manuscript:

MSL	Mexican Sign Language
LSTM	Long Short-Term Memory
CNN	Convolutional Neural Network
SLR	Sign Language Recognition
IMU	Inertial Measurement Unit
CNN-RNN	Convolutional Neural Network–Recurrent Neural Network
RGB	Red, Green, Blue
MLP	Multilayer Perceptron
UART	Universal Asynchronous Receiver–Transmitter
RGB-D	Red, Green, Blue—Depth
HUD	Heads-Up Display
ReLU	Rectified Linear Unit
